# Recovery and variation of the coastal fish community following a cold intrusion event in the Penghu Islands, Taiwan

**DOI:** 10.1371/journal.pone.0238550

**Published:** 2020-09-25

**Authors:** Hungyen Chen, Ching-Yi Chen, Kwang-Tsao Shao

**Affiliations:** 1 Department of Agronomy, National Taiwan University, Taipei, Taiwan; 2 National Museum of Marine Science and Technology, Keelung, Taiwan; 3 Biodiversity Research Center, Academia Sinica, Taipei, Taiwan; 4 Institute of Marine Biology, National Taiwan Ocean University, Keelung, Taiwan; 5 Center of Excellence for the Oceans, National Taiwan Ocean University, Keelung, Taiwan; Secretariat of the Pacific Community, NEW CALEDONIA

## Abstract

Knowledge of community resilience aids the development of strategies to mitigate the impacts of a disturbance. An extreme low-seawater temperature event in late January and February 2008 resulted in high fish mortality in the coastal waters of the Penghu Islands, Taiwan. In this study, we used underwater diving visual censuses to analyze fish communities at eight sampling stations along the coast of the Penghu Islands for seven years after the 2008 event. We evaluated community metrics, including species richness, abundance-weighted diversity, average thermal affinity, and average trophic level, and described the temporal variation in select dominant species abundances. Species richness and diversity of the communities required 53 months to reach a steady-state at the sampling stations following the cold water intrusion. The cold event initially reduced community thermal affinity, which then increased throughout the study period, reflecting the recovery of the community to baseline thermal conditions. The increased average trophic level after the cold event implied that the temperature shock decreased the proportion of lower trophic-level fishes. Average trophic level declined as the communities recovered from the disturbance, reflecting the recovery of the community to baseline species composition in terms of feeding habit. Our results suggest that functional diversity may require longer to recover than taxonomic diversity for communities in the Penghu Islands.

## Introduction

Stochastic extreme weather events drastically affect ecosystems and biological communities, as abiotic changes can result in extirpation of species or populations within a community [1]. Extreme weather events associated with climate change have increased since the 1990s, and they can lead to rapid changes in marine environments and organism dynamics [2, 3]. The impacts of disturbances triggered by sudden weather or climatic changes on marine ecosystems at the regional scale have been extensively studied. Higher seawater temperatures and altered hydrologic patterns caused by climatic changes have resulted in a decline in several fish populations [4–6]. Seawater warming alters the diversity patterns of demersal fish and temperate seaweeds, leading to shifts in the community structure, including decreasing diversity and the tropicalization of fish communities [7]. These changes in diversity are caused by proximate effects; for example, extreme seawater temperatures in Asia Pacific alter the spawning season and feeding grounds of marine fishes and decrease the hatching rate of captured fishes [8, 9]. Warming and cooling events in the upwelling zones can result in diminished fish population sizes and altered species structure, shifting towards lower trophic levels [10]. Extreme precipitation can affect the flow of ocean currents and modify the habitats of marine species [11]. Similarly, drought can induce seawater intrusion into an estuary, reducing the abundance of estuarine resident species and increasing the abundance of marine species [12].

Biotic communities, particularly those with high species diversity, exhibit ecological resilience which can buffer the negative effects of environmental changes [13]. A diverse community with greater functional variety is more likely to functionally compensate for the loss of some species under altered environmental conditions [13, 14]. In addition to monitoring species richness, which is the primary index for measuring community diversity, a comprehensive biodiversity investigation should assess the functional trait composition of the species in order to estimate functional diversity [15–18]. Functional diversity, introduced to evaluate redundancy and complementarity of species within a community [19], is related to ecological resistance and resilience, which are influenced by the traits of the dominant species in a community [18, 20, 21]. Because functional diversity elucidates evolutionary history, which is of conservation interest, the measures of functional diversity have been widely considered when establishing conservation approaches [18, 22, 23]. Monitoring variation in species and functional diversities within a community following perturbations from extreme weather events can help conservation biologists determine the severity of the perturbation and predict community recovery times [1, 24]. Moreover, even after diversity indices have returned to their original state, it is important to determine if any changes have occurred in the community species structure [25].

Extreme weather events caused by climatic change result in changes to marine environments which impact, fisheries, food security, and economic development [10, 26, 27]. The cold seawater intrusion that occurred in the Penghu Islands in early 2008 caused the death of resident fishes, including coral reef fishes and the fishes with low swimming capacity [28, 29]. The Penghu Islands in Taiwan are an archipelago of 92 islands and islets located in the southern Taiwan Strait. The average daily mean air temperature in Penghu is 23.5°C, with an average annual precipitation of 1,013 mm and 2,031 average annual sunshine hours between 1981 and 2010 [30]. Currents in the Taiwan Strait are complex: warm currents flow northward throughout the year, whereas the northeasterly monsoon lessens the strength of the warm current and drives cold currents from the north to northwest in winter [31]. From late January to February of 2008, high fish mortality was reported in the coastal waters of the Penghu Islands, due to an extreme low seawater temperature event [28, 32]. This incident, caused by an unusual intrusion of a cold front, significantly damaged marine aquaculture and wild fisheries, making it the most serious disaster in Penghu over the last few decades [28, 32]. A normal seawater temperature of 23.1°C was recorded at 3 m depth in Chinwan Inner Bay, Penghu, on January 12, 2008, which decreased to 11.7°C by February 15, 2008 [32]. Abnormally low and fluctuating temperatures persisted for one month and exceeded previous records from 2004 to 2007 [32]. The minimum seawater temperature recorded during this event was significantly below the critical thermal minimum (16.3°C) reported for some reef fishes [33].

Government reports on the cold intrusion event targeted only commercial fish species captured using various fishing methods, reported only annual governmental statistics [28, 29, 32, 34], and lacked data for all years following the cold water event [28, 32]. In this study, we surveyed coral reef fishes, which differ from the migratory fishes (including the vertically migratory deep-sea fishes and horizontally migratory high-priced fishes) captured in the government report. Most coral reef fishes are resident or semi-resident shallow water species. Their small home ranges (estimated to be between -1.11 and 6.88 (log_10_) m^2^ for 40 species [35]) render them more vulnerable to the impact of the cold intrusion event. Community stability and resilience are necessary to restore ecosystems and habitats to their original species compositions, rather than to an altered composition. However, studies on thermal affinity in relation to the trophic level of reef fish species remain scarce.

In this study, we analyzed temporal variations in the reef fish community for over seven years at eight sampling stations across the coastal waters of the Penghu Islands using underwater visual censuses. We aimed to determine the effects of the cold intrusion on the reef fish community and the recovery time for fishery resources in this area.

## Materials and methods

### Field surveys

Fish communities were surveyed at eight sampling stations in coastal waters of the Penghu Archipelago in Taiwan. Two stations each were established at the inner (stations I1: N23°32.19′, E119°33.31′ and I2: N23°33.06′, E119°33.03′), northern (N1: N23°47.13′, E119°35.40′ and N2: N23°44.32′, E119°35.34′), eastern (E1: N23°39.14′, E119°40.03′ and E2: N23°41.22′, E119°38.57′), and southern (S1: N23°23.41′, E119°29.20′ and S2: N23°22.09′, E119°32.32′) areas of the archipelago. The water depth at the inner, northern, eastern, and southern sampling stations was 3–8, 5–7, 5–11, and 4–9 m, respectively. The sea floor was a mixture of coral reefs, rocks, and muddy and sandy patches at the central and southern stations; a mixture of coral reefs, rocks, and sandy patches at the eastern stations; and a mixture of rocks and sandy patches at the northern stations. We did not include the western region of the Penghu Islands, because the coastal waters around the western area are more than 10 m in depth, which is sufficiently deep to shelter fishes from the cold intrusion event.

Fish community surveys were conducted using underwater visual census. Fish species abundances were recorded by two divers along a transect line at each sampling station (50 m × 2 m area). Fishes were identified by the same senior laboratory members, Miss Ching-Yi Chen (20 years of experience) and Mister Jeng-I Tsai (15 years of experience), throughout the study period for consistency. Two-20 minute surveys were conducted at each station per visit. An underwater video was recorded for validating the exact number of observed fishes later in the laboratory. The surveys were conducted in April, May, and June 2008, in May and September 2009 and 2010, and in September 2013 and 2014, because the weather conditions at Penghu Islands are suitable to divers for diving from May to October (seawater temperature of 22–28°C at 2–10 m depth, S1 Table). The abundances of each species recorded during the two daily surveys at each station were summed to reduce zero data for analyses. All field work was conducted with permission from the Fishery Administration, Council of Agriculture, Taiwan.

### Diversity indices and statistical models

We quantified changes in the biodiversity of fish assemblages using the traditional approaches of species richness and abundance-weighted diversity. Species richness was calculated as the number of species (*n*). The species diversity was calculated using the “diversity” function (to calculate Shannon’s entropy [36]) in the “vegan” package [37] in R 3.0.2 software [38], which incorporates the relative abundance (number of individuals) of each species per sample. This index is calculated as the proportion of species *i* relative to the total number of species (*P*_*i*_) multiplied by the natural logarithm of the species proportion (ln(*P*_*i*_)).

We further analyzed trends in species traits to ascertain how changes in species composition corresponded with traits present in the community. We selected the lower percentile of the realized temperature distribution (estimated lower temperature [39]) for each species in a community to represent thermal affinity [14]. The average thermal affinity across all species present in the community allows for comparing the species composition with traits of different fundamental thermal niches in the communities by assigning a low temperature to each species in the community. The average trophic level [39] across all species present in the community was calculated to measure temporal variations in species composition with traits of different trophic groups in the communities. The relative abundance of each species was ignored to calculate these indices, so that each species contributed equally. Data for the estimated lower temperature and trophic level for each species were obtained from FishBase [39] (S2 Table). The collected fish species and their lower temperatures and trophic levels are listed in S2 Table.

Trends of species richness and diversity, average thermal affinity, and average trophic level against time were analyzed using linear or quadratic mixed effects regression models including station as the random factor, and the best model was selected based on the Akaike information criterion [40, 41]. We defined the recovery time (the time needed for the community to reach a steady state at a sampling area) as the extremum (maximum or minimum number) of the quadratic model.

## Results

### Recovery of species richness and diversity

**Fig 1:**
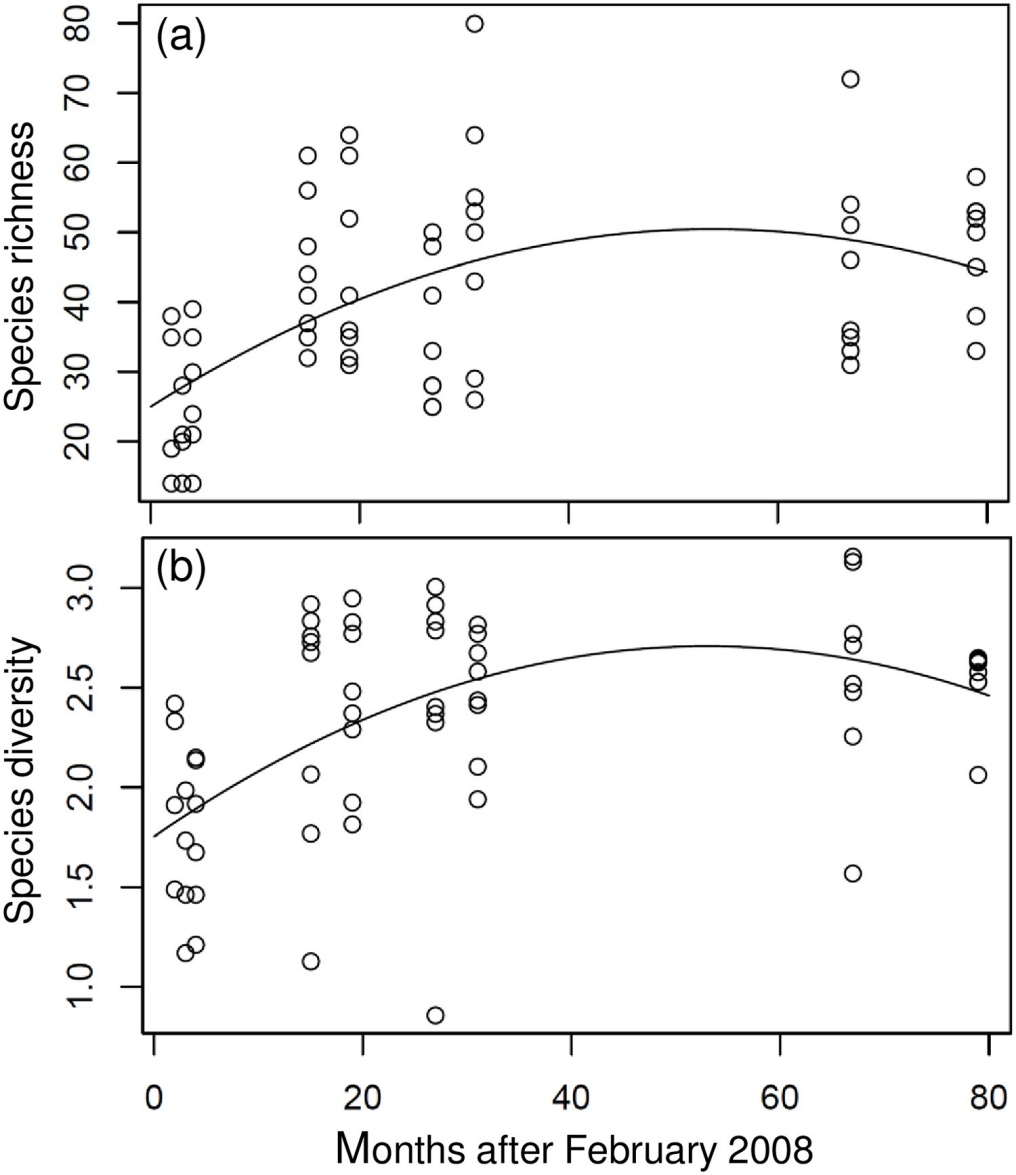
Temporal variation in (a) species richness and (b) species diversity in the community at all of the sampling stations. Open circles represent surveys, black curve represents the quadratic regression curve.

It is evident from Fig 1 that both species richness and diversity could be fitted using a quadratic regression model with negative quadratic term (Tables 1 and 2). These indices increased from April 2008 and peaked 53 months after the disturbance event (Table 3 and Fig 1). The estimated intercept and maxima were 24.98 and 50.44, respectively, for species richness, and 2.708 and 1.753, respectively, for species diversity (Tables 2 and 3). These results imply that 53 months were required for the recovery of both species richness and diversity at the Penghu Islands after the cold intrusion event. Species richness and diversity increased by 101.9% and 54.4%, respectively, after the cold event.

**Table 1:** Comparison of the Akaike information criterion for the regression models of the indices against time.

Index	Null model	Linear model	Quadratic model
Species richness	511	502	496
Species diversity	101	93.0	86.2
Thermal niche	50.7	38.2	33.6
Trophic level	-84.2	-95.4	-98.3

**Table 2:** Estimates from the quadratic regression models of the indices against time and the coefficient of determination.

Index	Intercept		Linear term		Quadratic term		Coefficient of determination
	Estimate	SE	Estimate	SE	Estimate	SE	
Species richness	24.98	3.785	0.948	0.263	-0.009	0.003	0.262
Species diversity	1.753	0.139	0.036	0.010	-0.001	0.001	0.266
Thermal affinity	23.55	0.091	0.022	0.006	-0.001	0.001	0.288
Trophic level	3.369	0.031	-0.001	0.001	0.001	0.001	0.254

SE indicates standard error.

**Table 3:** Extrema and their corresponding time in the quadratic regression models of the indices against time.

Index	Extrema		Number of months to reach extrema
	Maxima	Minima	
Species richness	50.44	-	53
Species diversity	2.708	-	53
Thermal affinity	24.18	-	57
Trophic level	-	3.174	58

### Variations in the number of species and individuals of dominant fishes

**Fig 2:**
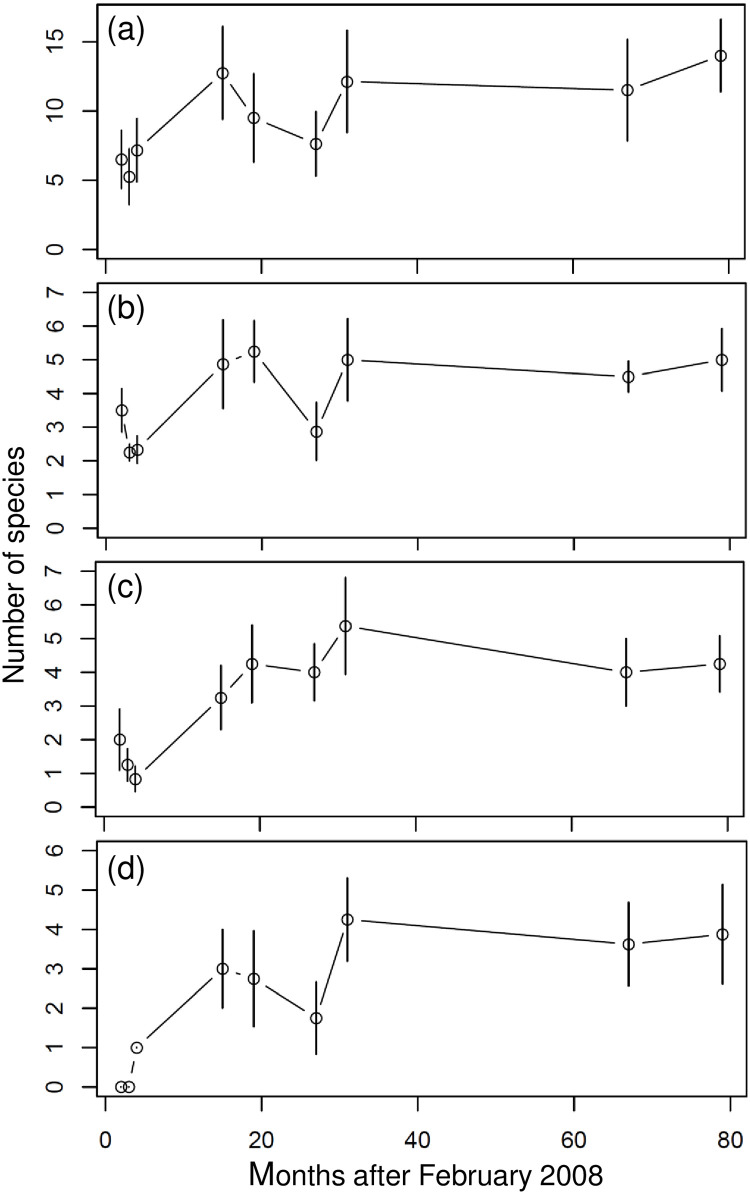
Temporal variation in number of species present in surveys from dominant families. (a) Labridae. (b) Pomacentridae. (c) Chaetodontidae. (d) Scaridae. Open circles represent the mean at all of the sampling stations, and the length of the vertical bars represent one standard deviation.

**Fig 3:**
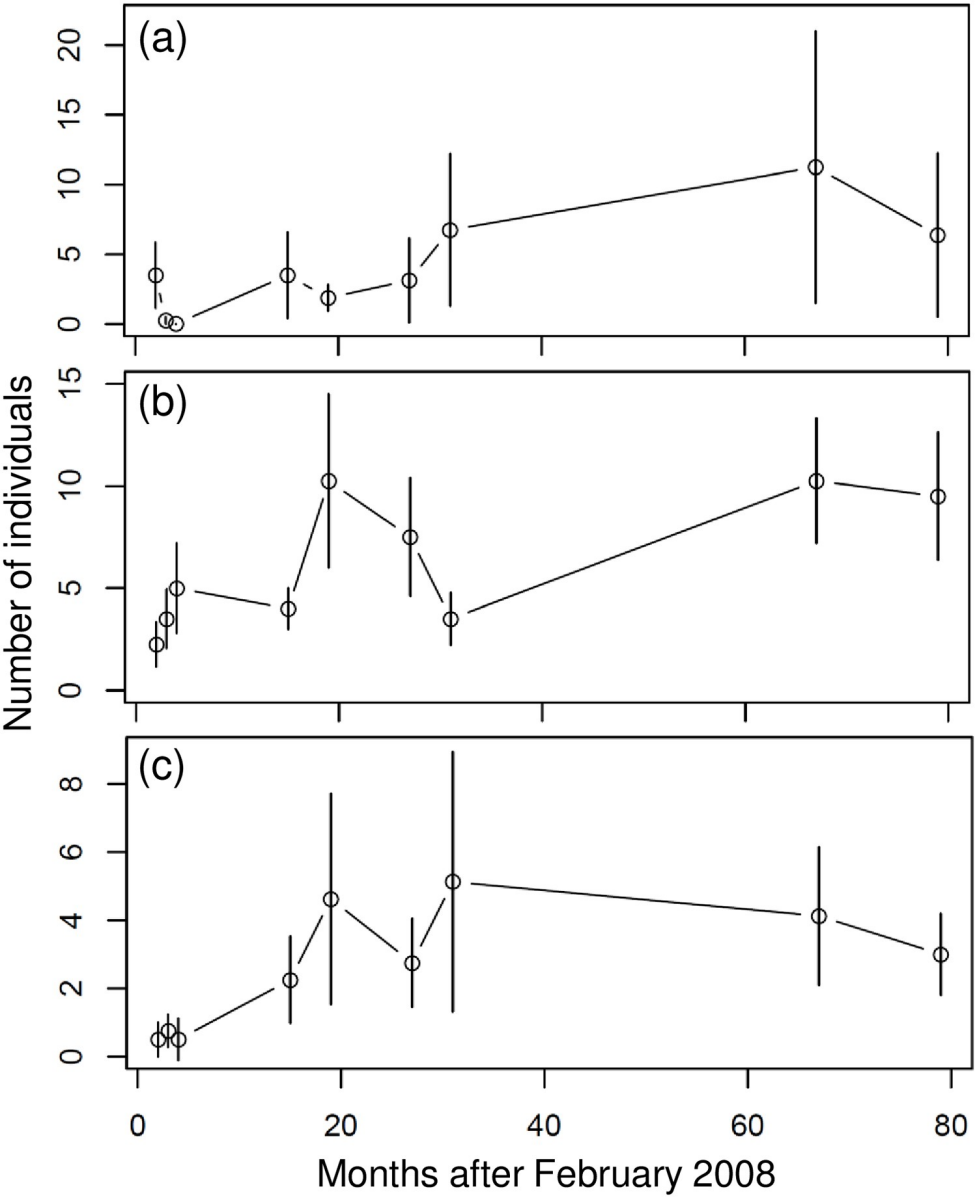
Temporal variation in number of individuals of dominant species. (a) *Chaetodon octofasciatus*. (b) *Epinephelus quoyanus*. (c) *Plectropomus leopardus*. Open circles represent the mean at all of the sampling stations, and the length of the vertical bars represent one standard deviation.

Labridae (62 of 322 species; 19.3%), Pomacentridae (9.3%), Chaetodontidae (6.8%), and Scaridae (5.6%) were the four most species-rich fish families at the Penghu Islands. On average, less than eight Labridae species (wrasse, Fig 2a) were recorded per sample at all stations in 2008, and less than four species each were recorded for Pomacentridae (damselfish, Fig 2b), Chaetodontidae (butterflyfish, Fig 2c), and Scaridae (parrotfish, Fig 2d) at all stations in April 2008. The average number of species within these four families increased considerably after 2008 until 2010, and remained steady between 2010 and 2014 (Fig 2). Abundances of dominant (most frequently observed) species also changed throughout the survey period. Less than five individuals of *Chaetodon octofasciatus* (eight-banded butterflyfish, Fig 3a), *Epinephelus quoyanus* (longfin grouper, Fig 3b), and *Plectropomus leopardus* (coral trout, Fig 3c) were recorded on average per sample at all stations in 2008, but increased after 15 months.

### Variations in functional diversities

**Fig 4:**
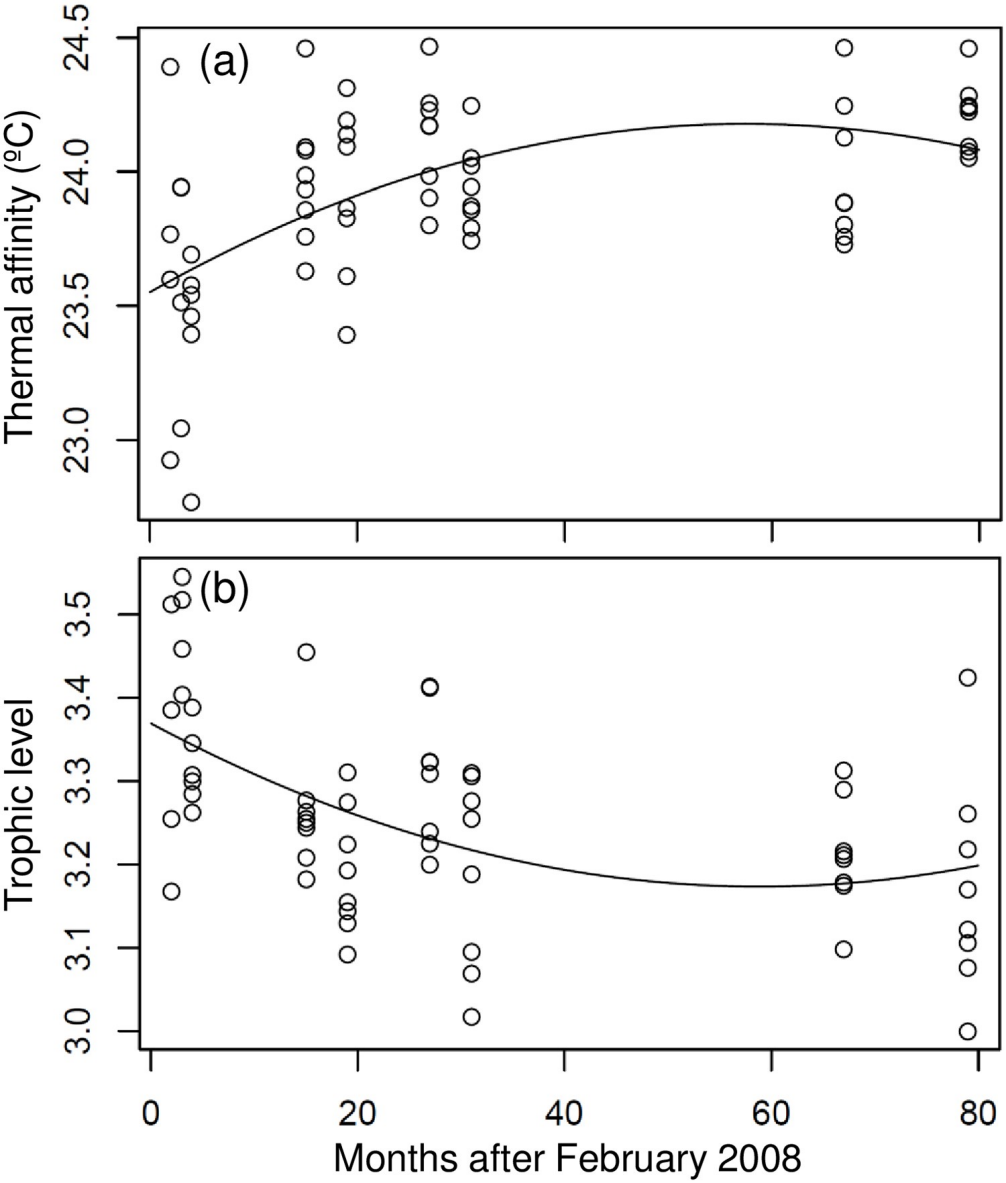
Temporal variation in (a) average thermal affinity and (b) average trophic level across all species present in the community at all of the sampling stations. Open circles represent surveys, and black curve represents the quadratic regression curve.

It is evident from Fig 4 that average thermal affinity and average trophic level could be fitted using a quadratic regression model with negative and positive quadratic terms, respectively (Tables 1 and 2). The community thermal affinity, measured as the average thermal affinity across all species in the community, increased after April 2008 and peaked 57 months after the cold disturbance event (Table 3 and Fig 4a). The estimated intercept and maxima for average thermal affinity were 23.55 and 24.18, respectively (Tables 2 and 3). The difference between the intercept and maxima was 0.63, implying that 57 months were required for the average thermal affinity to increase by 0.63°C at all sampling stations after the cold intrusion event. The cold intrusion event initially reduced average thermal affinity, which then increased as communities recovered from the temperature shock of 2008, reflecting the recovery of the community to normal baseline thermal conditions. The average trophic level across all species present in the community decreased after April 2008 and reached a minimum 58 months after the cold event (Table 3 and Fig 4b). The estimated intercept and maxima for average trophic level were 3.369 and 3.174, respectively, with a difference of -0.195 (Tables 2 and 3). The increase in average trophic level after the cold event implied that the temperature shock of 2008 decreased the proportion of fishes feeding at lower trophic levels, such as some herbivorous fishes. The average trophic level declined as communities recovered from the disturbance, reflecting the recovery of the community to normal species composition in terms of feeding habit. These results imply that functional diversity may take longer to recover than taxonomic diversity for communities in the Penghu Islands.

## Discussion

In the La Niña winter of 2008, the wind speed increased sharply on January 26 and peaked in February, remaining at that level for more than three weeks [28]. The strong winds resulted in a southeasterly cold current in the southern Taiwan Strait, which resulted in a significant decrease in seawater temperature [28]. This cold front greatly damaged aquaculture, resulting in a 60% reduction in the coastal fishery output of the Penghu Islands in 2008 [32]. In total, the cold event killed more than 73 t of marine fishes and 1,500 t of aquaculture fishes in Penghu [28, 29]. Dead individuals of more than 183 resident fish species belonging to 58 families were recorded on the beaches, including several large, highly-priced species such as wrasse, grouper, and parrotfish [32]. Because these highly-priced species are collected intensively by local residents, their losses have likely been underestimated [32]. The results of this study fit in the context of global climatic change by providing useful information about how extreme weather events affected marine fishes and how much time the recovery of fishery resources is needed from the cold intrusion in the Penghu Islands.

The effects of cold events on fisheries have been reported in several studies [33, 42–44]. Lower seawater temperatures during the La Niña years have been shown to decrease the abundance of several reef fishes in the eastern Pacific Ocean [33]. Similarly, an extreme cold event caused widespread mortality of the common snook in South Florida [44]. Cold water intrusions often result in turbid waters, which may reduce the feeding efficiency of nonmigratory fishes or cause them to disperse from their natural habitats [42]. Negative effects of low seawater temperatures have also been documented for marine invertebrates, including the spiny lobster [45], mud crab [46], and sea cucumber [47]. Lower temperatures may also affect immune functionality, and lead to energy and nutrition loss in fishes because of reduced feeding activities [43]. Thus, the extremely cold water in February 2008 may have limited the feeding activities of resident and warm-water fishes in the Penghu Islands [48].

Mass mortalities of coastal fishes caused by low water temperature in the coastal waters of the Penghu Islands have previously been reported in 1934 and 1977, when the air temperatures fell below 9°C [49]. The present study monitored the species richness and abundance after the cold-water intrusion event of February 2008; however, no fish censuses are available before the intrusion. Our results show that at least 45 months were required for the communities to reach a steady state following the cold water front. To estimate the extent of the effects of this cold intrusion on species richness, we compared the results of 2008 with those of investigations conducted at the same sampling stations in 1992 or 1993 [50–53]. In 1992/93, the average species richness was 55.8 (sd = 14.1) for stations I1, I2, N2, S1, and S2, whereas no data were available for the eastern area [50–53]. The lowest species richness recorded in 2008 was 44.4% (sd = 12.8%) of that recorded in 1992/93. The highest average species richness between 2008 and 2014 was 55.4 (sd = 12.0) for the five stations, which was 100.2% (sd = 15.3%) of that recorded in 1992/93. Compared with data from 1992/93, the cold intrusion most strongly affected species richness in the central area, which was restored to its 1992/93 status or greater from 2010 to 2013 at all sampling stations. In contrast, the species richness in the south was only slightly affected when compared with the central and northern areas. However, more metrics are needed to assess the recovery of an ecosystem from a disturbance [54].

Our results indicate that only the species composition of samples collected in 2008 differed from that in other years. This difference may be attributed to changes in dominant species abundances, environment, or habitat after the cold event. Few species were observed after the stochastic cold event, but included small (2–3 cm) gobies (Gobiidae), black wrasse *Halichoeres melanochir*, fire-tailed dottyback *Labracinus cyclophthalmus*, rabbitfish *Siganus fuscescens*, and longfin grouper *Epinephelus quoyanus*. Abundances of traditionally dominant species of the Penghu Islands, such as the eight-banded butterflyfish *C*. *octofasciatus* and bullethead parrotfish *Chlorurus sordidus*, were also extremely low. Wrasses, butterflyfishes, and damselfishes are coral reef species occupying the shallow waters of the Penghu Islands and are important aquarium fishes. The three large-bodied fishes observed after the cold event of 2008, i.e., the green snapper *Lethrinus nebulosus*, blackspot tuskfish *Choerodon schoenleinii*, and leopard coral trout *P*. *leopardus*, prefer water depths of 25 m, suggesting a stronger effect of the cold intrusion event on shallow-water fishes.

The results showed that the average thermal affinity of the species in the community changed over the course of the study (i.e., since the cold intrusion event). This event also affected the coral reefs along the coasts of the Penghu Islands [32]. Widespread bleaching of staghorn corals (*Acropora formosa*) was observed near the coast in May 2008, following the decrease in water temperature in late January 2008. Many butterflyfishes are mutualistic with coral reefs [55]; thus, the time required for the recovery of species richness and species composition of the community may vary depending on the growth status of coral reefs. It is also likely that the increase in herbivores could be caused by the more abundant algal beds after coral bleaching [56, 57]. In addition to corals, death of macroinvertebrates, including echinoderms, crustaceans, and mollusks, was reported in the surrounding seas [32]. Our results demonstrate a change in the species composition in terms of trophic function, indicated by a decrease in the average trophic level, despite an observed recovery in species richness. Although the cardinalfishes are not economically valuable, they are important food sources for carnivorous fishes such as groupers (Serranidae), snappers (Lutjanidae), and hardtails (Carangidae). A lack of cardinalfishes in the food web would decrease the abundance of carnivorous fishes, and may thus alter the ecosystem structure [58, 59]. An increase in the herbivores in the fish community may be a potential mechanism of functional community change [14, 60]. This study provides a case study of how the fish assemblages varied and recovered after a cold water intrusion, however, the consistent long-term monitoring is still necessary to elucidate the entire influence on the ecosystem [61–63].

## Conclusions

In summary, over the seven years following an extreme marine climatic event, distinct trends were observed in the species and traits composition of fish communities across eight sampling stations in the Penghu Islands. The communities were observed to be resilient in terms of species richness and diversity. These results may depend on the recovery of marine habitats, patterns, and availability of fish larval recruits, local ocean currents, and marine environmental changes. This study demonstrates the long-term effects of extreme weather events on marine fish communities, and highlights the need for regular monitoring of communities for predicting and mitigating the subsequent negative effects, particularly under climate change. Knowledge of the recovery time of natural ecosystems after cold intrusions is required for ecosystem management. Therefore, it is recommended that the government conduct studies on the scale and duration of compensation for recovery, and the time required for monitoring. Our results provide valuable information regarding the duration of the effects of the cold intrusion on the composition and function of fish populations.

## Supporting information

S1 Table Water temperature at all sampling stations over the course of the study. (DOCX)

S2 Table List of collected fish species with information on lower temperature and trophic level. (DOCX)

## References

[1] Scheffer M, Carpenter S, Foley JA, Folke C, Walker B. Catastrophic shifts in ecosystems. Nature. 2001; 413: 591–596. 10.1038/35098000 11595939

[2] Easterling DR, Meehl GA, Parmesan C, Chagnon SA, Karl T, Mearns LO. Climate extremes: observation, modeling and impacts. Science. 2000; 289: 2068–2074. 10.1126/science.289.5487.2068 11000103

[3] Walther G-R, Post E, Convey P, Menzel A, Parmesan C, Beebee TJC, et al Ecological responses to recent climate change. Nature. 2002; 416: 389–395. 10.1038/416389a 11919621

[4] Yatsu A, Watanabe T, Ishida M, Sugisaki H, Jacobson LD. Environmental effects on recruitment and productivity of Japanese sardine *Sardinops melanostictus* and chub mackerel *Scomber japonicus* with recommendations for management. Fish. Oceanogr. 2005; 14: 263–78.

[5] Lloret J, Marin A, Marin-Guirao L. Is coastal lagoon eutrophication likely to be aggravated by global climate change? Estuar Coast Shelf Sci. 2008; 78: 403–412.

[6] Knutson TR, McBride JL, Chan J, Emanuel K, Holland G, Landsea C, et al Tropical cyclones and climate change. Nat Geosci. 2010; 3: 157–163.

[7] Wernberg T, Smale DA, Tuya F, Thomsen MS, Langlois TJ, de Bettignies T, et al An extreme climatic event alters marine ecosystem structure in a global biodiversity hotspot. Nat Clim Change. 2012; 3: 78–82.

[8] Lin C, Ning X, Su J, Lin Y., Xu B. Environmental changes and the responses of the ecosystems of the Yellow Sea during 1976–2000. J Mar Syst. 2005; 55: 223–234.

[9] FAO. Environmental impact assessment and monitoring in aquaculture. FAO fisheries and aquaculture technical paper no. 527. Rome: 2009. p 648.

[10] Allison EH, Perry AL, Adger WN, Badjeck MC, Brown K, Conway D, et al Vulnerability of national economies to the impacts of climate change on fisheries. Fish. 2009; 10: 173–196.

[11] Whitehead PG, Wilby RL, Battarbee RW, Kernan M, Wade AJ. A review of the potential impacts of climate change on surface water quality. Hydrolog Sci J. 2009; 54: 101–123.

[12] Martinho F, Leitão R, Viegas I, Dolbeth M, Neto JM, Cabral H, et al The influence of an extreme drought event in the fish community of a southern Europe temperate estuary. Estuar Coast Shelf Sci. 2007; 75: 537–546.

[13] Bernhardt JR, Leslie HM. Resilience to climate change in coastal marine ecosystems. Annu Rev Mar Sci. 2013; 5: 371–392. 10.1146/annurev-marine-121211-172411 22809195

[14] Bates AE, Barrett NS, Stuart-Smith RD, Holbrook NJ, Thompson PA, Edgar GJ. Resilience and signatures of tropicalization in protected reef fish communities. Nat Clim Change. 2014; 4: 62–67.

[15] Petchey OL, Gaston KJ. Functional diversity (FD), species richness and community composition. Ecol Lett. 2002; 5: 402–411.

[16] Chen H, Shao K-T, Kishino H. Phylogenetic skew: an index of community diversity. Mol Ecol. 2015; 24: 759–770. 10.1111/mec.13064 25580733

[17] Chen H, Kishino H. Global pattern of phylogenetic species composition of shark and its conservation priority. Ecol Evol. 2015; 5: 4455–4465. 10.1002/ece3.1724 26819704 PMC4667821

[18] Chen H, Nagai S, Kishino H. Assessment of the network of protected areas for birds in Taiwan with regard to functional and phylogenetic diversity. Pacific Conserv Biol. 2016; 22: 61–71.

[19] Díaz S, Cabido M. Vive la difference: plant functional diversity matters to ecosystem processes. Trend Ecol Evol. 2001; 16: 646–655.

[20] Lepš J, Osbornová-Kosinová J, Rejmánek M. Community stability, complexity and species life-history strategies. Vegetatio. 1982; 50: 53–63.

[21] MacGillivray CW, Grime JP, the Integrated Screening Programme (ISP) Team. Testing predictions of the resistance and resilience of vegetation subjected to extreme events. Func Ecol. 1995; 9: 640–649.

[22] Mace GM, Gittleman JL, Purvis A. Preserving the tree of life. Science. 2003; 300: 1707–1709. 10.1126/science.1085510 12805539

[23] Knapp S, Kühn I, Schweiger O, Klotz S. Challenging urban species diversity: contrasting phylogenetic patterns across plant functional groups in Germany. Ecology Letters. 2008; 11: 1054–1064. 10.1111/j.1461-0248.2008.01217.x 18616547

[24] Lake PS. Ecological effects of perturbation by drought in flowing waters. Freshwater Biol. 2003; 48: 1161–1172.

[25] Holling CS. Resilience and stability of ecological systems. Annu Rev Ecol Syst. 1973; 4: 1–23.

[26] Badjeck M-C, Allison EH, Halls AS, Dulvy NK. Impacts of climate variability and change on fishery-based livelihoods. Mar Policy. 2010; 34: 375–383.

[27] Sumaila R, Cheung WWL, Lam VWY, Pauly D, Herrick S. Climate change impacts on the biophysics and economics of world fisheries. Nat Clim Change. 2011; 1: 449–456.

[28] Chang Y, Lee K-T, Lee M-A, Lan K-W Satellite observation on the exceptional intrusion of cold water in the Taiwan Strait. Terr Atmos Ocean Sci. 2009; 20: 661–669.

[29] Chang Y, Lee M-A, Lee K-T, Shao K-T. Adaptation of fisheries and mariculture management to extreme oceanic environmental changes and climate variability in Taiwan. Mar Policy. 2013; 38: 476–482.

[30] Central Weather Bureau. 2017. http://www.cwb.gov.tw/. Accessed 11 Nov 2017

[31] Jan S, Wang J, Chern C-S, Chao S-Y. Seasonal variation of the circulation in the Taiwan Strait. J Mar Syst. 2002; 35: 249–268.

[32] Hsieh H-J, Hsien Y-L, Tsai W-S. Tropical fishes killed by the cold. Coral Reefs. 2008; 27: 599.

[33] Mora C, Ospina AF. Experimental effect of cold, La Niña temperatures on the survival of reef fishes from Gorgona Island (eastern Pacific Ocean). Mar Biol. 2002; 141: 789–793.

[34] Lee M-A, Lee K-T, Shao K-T, Tzeng J-J, Jeng M-S, Jan M-S. Further investigations of fishery resources and its precaution system related to cold water intrusion in Peng-Hu waters. Technic project report of Fishery Agency, 99–8.5.2-F1(4). 2010 p 267.

[35] Nash KY, Welsh JQ, Graham NAJ, Bellwood DR. Home range allometry in coral reef fishes: comparison to other vertebrates, methodological issues and management implications. Oecologia. 2015; 177: 73–83. 10.1007/s00442-014-3152-y 25424157

[36] Shannon CE. A mathematical theory of communication. Bell Syst Tech J. 1948; 27: 379–423.

[37] Oksanen J, Kindt R, Legendre P, O’Hara RB. vegan: Community Ecology Package. R package version 1.8–3. 2006.

[38] R Core Team. R: A language and environment for statistical computing. R Foundation for Statistical Computing, Vienna, Austria 2013 https://www.R-project.org/. Accessed 23 Dec 2013.

[39] Froese R, Pauly D. Editors. FishBase. World Wide Web electronic publication. www.fishbase.org, version 02/2019. 2019. Accessed 23 Feb 2019.

[40] Akaike H. A new look at the statistical model identification. IEEE T Automat Contr. 1974; 19: 716–723.

[41] Chen H. The spatial patterns in long-term temporal trends of three major crops’ yields in Japan. Plant Prod Sci. 2018; 21: 177–185.

[42] Wroblewski JS, Richman JG. The non-linear response of plankton to wind mixing events—implications for survival of larval northern anchovy. J Plankton Res. 1987; 9: 103–123.

[43] Le Morvan C, Troutaud D, Deschaux P. Differential effects of temperature on specific and nonspecific immune defences in fish. J Exp Biol. 1998; 201: 165–168. 9405298 10.1242/jeb.201.2.165

[44] Stevens PW, Blewett DA, Boucek RE, Rehage JS, Winner BL, Young JM, et al Resilience of a tropical sport fish population to a severe cold event varies across five estuaries in southern Florida. Ecosphere. 2016; 7: e01400.

[45] Morikawa Y, Arakawa H, Koike T. Effects of water temperature on diurnal feeding activity of Japanese spiny lobster *Pannlirus japonicus*. Nippon Suisan Gakk. 2000; 66: 791–798 (in Japanese).

[46] Hill BJ. Effects of temperature on feeding and activity in the crab *Scylla serrata*. Mar Biol. 1980; 59: 189–192.

[47] Wolkenhauer SM. Burying and feeding activity of adult *Holothuria scabra* (Echinodermata: Holothuroidea) in a controlled environment. SPC Beche de Mer Inform Bull. 2008; 27: 25–28.

[48] Lee M-A, Yang Y-C, Shen Y-L, Chang Y, Tsai W-S, Lan K-W, et al Effects of an unusual cold-water intrusion in 2008 on the catch of coastal fishing methods around Penghu Islands, Taiwan. Terr Atmos Ocean Sci. 2014; 25: 107–120.

[49] Tang H-G. The situations and reviews of the mass mortalities due to freeze during winter time in Pescadores. China Fish Mon. 1978; 302: 24–26 (in Chinese).

[50] Chen C-C, Shao K-T, Liu C-L, Chen C-C, Yu S-P, Chen Y-C, et al Investigation report on marine biological resources in the eastern seas of Penghu Islands. Penghu National Scenic Area Administration, Tourism Bureau. 1992.

[51] Chang K-S, Jang Y-M, Chen C-H, Jang Z-G, Dai C-F, Jan M-S. Investigation report on marine biological resources in the northern seas of Penghu Islands. Penghu National Scenic Area Administration, Tourism Bureau. 1992.

[52] Chang K-S, Yang H-N, Chen C-H, Jang Z-G, Dai C-F, Jan M-S. Investigation report on marine biological resources in the inner seas of Penghu Islands. Penghu National Scenic Area Administration, Tourism Bureau. 1993.

[53] Fang S-C, Mo S-C, Chen H-Y, Song K-Y, Liu L-L. Investigation report on marine biological resources in the southern seas of Penghu Islands. Penghu National Scenic Area Administration, Tourism Bureau. 1993.

[54] Peterson G, Allen CR, Holling CS. Ecological resilience, biodiversity, and scale. Ecosystems. 1998; 1: 6–18.

[55] Roberts CM, Ormond RFG. Butterflyfish social behaviour, with special reference to the incidence of territoriality: a review. Environ Biol Fish. 1992; 34: 79–93.

[56] Boyer KE, Fong P, Armitage AR, Cohen RA. Elevated nutrient content of tropical macroalgae increases rates of herbivory in coral, seagrass, and mangrove habitats. Coral Reefs. 2004; 23: 530–538.

[57] Vergés A, Doropoulos C, Malcolm HA, Skye M, Garcia-Pizá M, Marzinelli EM, et al Long-term empirical evidence of ocean warming leading to tropicalization of fish communities, increased herbivory, and loss of kelp. PNAS. 2016; 113: 13791–13796. 10.1073/pnas.1610725113 27849585 PMC5137712

[58] Sih A, Crowley P, McPeek M, Petranka J, Strohmeier K. Predation, competition, and prey communities: a review of field experiments. Annu Rev Ecol Syst. 1985; 16: 269–311.

[59] Heath MR. Changes in the structure and function of the North Sea fish foodweb, 1973–2000, and the impacts of fishing and climate. ICES J Mar Sci. 2005; 62: 847–868.

[60] Sala E, Kizilkaya Z, Yildirim D, Ballesteros E. Alien marine fishes deplete algal biomass in the eastern Mediterranean. PLoS ONE. 2011; 6: e17356 10.1371/journal.pone.0017356 21364943 PMC3043076

[61] Chen H, Liao Y-C, Chen C-Y, Tsai J-I, Chen L-S, Shao K.-T. Long-term monitoring dataset of fish assemblages impinged at nuclear power plants in northern Taiwan. Sci Data. 2015; 2: 150071 10.1038/sdata.2015.71 26647085 PMC4672678

[62] Chen H, Chen C-Y. Shao K-T. Time series dataset of fish assemblages near thermal discharges at nuclear power plants in northern Taiwan. Sci Data. 2018; 5: 180055. 29737979 10.1038/sdata.2018.85 PMC5944904

[63] Ho L-T, Wang S-C, Shao K-T, Chen I-S, Chen H. A long-term monitoring dataset of fish assemblages in rocky tidepools on the northern coast of Taiwan. Sci Data. 2020; 7: 84 10.1038/s41597-020-0425-7 32152315 PMC7063039

