# Supplementary material for: Recovery and variation of the coastal fish community following a cold intrusion event in the Penghu Islands, Taiwan
Source: PLoS One. 2020 Sep 25;15(9):e0238550. doi: 10.1371/journal.pone.0238550 (PMC7518628; doi:10.1371/journal.pone.0238550)
Supplement: S1 Table — (DOCX) [file pone.0238550.s001.docx]

**Supporting information**

**S1 Table.** **Water temperature at all sampling stations over the course of the study.**

| Water Temperature (ºC) | 2008 | | | 2009 | | 2010 | | 2013 | 2014 |
| --- | --- | --- | --- | --- | --- | --- | --- | --- | --- |
| Station | April | May | June | May | September | May | September | September | September |
| I1 | 23 | 25 | 25 | 25 | 28 | 24 | 28 | 28 | 28 |
| I2 | 24 | 25 | 25 | 25 | 28 | 24 | 27 | 28 | 27 |
| N1 | - | 24 | 25 | 24 | 27 | 23 | 27 | 27 | 27 |
| N2 | - | 24 | 25 | 24 | 27 | 24 | 28 | 27 | 27 |
| E1 | - | - | 24 | 24 | 26 | 23 | 26 | 27 | 27 |
| E2 | - | - | 23 | 24 | 27 | 22 | 27 | 27 | 27 |
| S1 | 22 | - | - | 24 | 26 | 24 | 26 | 26 | 26 |
| S2 | 22 | - | - | 24 | 26 | 23 | 25 | 25 | 25 |
