# Supplementary material for: Recovery and variation of the coastal fish community following a cold intrusion event in the Penghu Islands, Taiwan
Source: PLoS One. 2020 Sep 25;15(9):e0238550. doi: 10.1371/journal.pone.0238550 (PMC7518628; doi:10.1371/journal.pone.0238550)
Supplement: S2 Table — (DOCX) [file pone.0238550.s002.docx]

**Supporting information**

**S2 Table. List of collected fish species with information on lower temperature and trophic level.**

| Family | Species | Lower Temperature | Trophic Level |
| --- | --- | --- | --- |
| Acanthuridae | *Acanthurus olivaceus* | 24.5 | 2.3 |
| Acanthuridae | *Ctenochaetus striatus* | 24.6 | 2.0 |
| Acanthuridae | *Zebrasoma scopas* | 24.6 | 2.0 |
| Acanthuridae | *Acanthurus nigricans* | 24.5 | 2.0 |
| Acanthuridae | *Acanthurus pyroferus* | 25.0 | 2.0 |
| Acanthuridae | *Acanthurus triostegus* | 23.4 | 2.8 |
| Acanthuridae | *Naso brevirostris* | 24.3 | 2.2 |
| Acanthuridae | *Naso annulatus* | 23.5 | 2.1 |
| Acanthuridae | *Naso unicornis* | 23.4 | 2.2 |
| Acanthuridae | *Acanthurus bariene* | 24.5 | 2.0 |
| Acanthuridae | *Zebrasoma veliferum* | 24.8 | 2.0 |
| Acanthuridae | *Acanthurus thompsoni* | 24.6 | 3.6 |
| Acanthuridae | *Acanthurus lineatus* | 24.7 | 2.0 |
| Acanthuridae | *Acanthurus nigrofuscus* | 24.6 | 2.0 |
| Acanthuridae | *Prionurus scalprum* | 17.7 | 2.0 |
| Acanthuridae | *Acanthurus xanthopterus* | 23.3 | 2.9 |
| Acanthuridae | *Acanthurus dussumieri* | 20.3 | 2.0 |
| Apogonidae | *Apogon coccineus* | 24.7 | 3.5 |
| Apogonidae | *Archamia fucata* | 25.1 | 3.5 |
| Apogonidae | *Cheilodipterus quinquelineatus* | 24.9 | 3.9 |
| Apogonidae | *Ostorhinchus aureus* | 24.7 | 3.5 |
| Apogonidae | *Ostorhinchus endekataenia* | 21.1 | 3.5 |
| Apogonidae | *Ostorhinchus novemfasciatus* | 25.0 | 4.0 |
| Apogonidae | *Ostorhinchus properuptus* | 22.0 | 3.3 |
| Apogonidae | *Cheilodipterus macrodon* | 24.7 | 4.0 |
| Apogonidae | *Siphamia majimai* | 24.7 | 3.4 |
| Apogonidae | *Ostorhinchus cookii* | 23.2 | 3.5 |
| Apogonidae | *Ostorhinchus pleuron* | 25.2 | 3.6 |
| Apogonidae | *Ostorhinchus holotaenia* | 24.8 | 3.4 |
| Apogonidae | *Apogon doederleini* | 20.0 | 3.6 |
| Atherinidae | *Hypoatherina woodwardi* | - | 3.4 |
| Aulostomidae | *Aulostomus chinensis* | 24.2 | 4.2 |
| Balistidae | *Pseudobalistes flavimarginatus* | 24.7 | 2.8 |
| Balistidae | *Sufflamen chrysopterum* | 24.8 | 3.5 |
| Blenniidae | *Aspidontus taeniatus* | 25.1 | 3.8 |
| Blenniidae | *Entomacrodus decussatus* | 25.4 | 2.0 |
| Blenniidae | *Entomacrodus lighti* | 25.1 | 2.0 |
| Blenniidae | *Exallias brevis* | 24.7 | 4.3 |
| Blenniidae | *Meiacanthus grammistes* | 26.4 | 3.4 |
| Blenniidae | *Plagiotremus tapeinosoma* | 23.9 | 3.8 |
| Blenniidae | *Istiblennius dussumieri* | 23.1 | 2.0 |
| Blenniidae | *Petroscirtes breviceps* | 25.0 | 2.1 |
| Blenniidae | *Plagiotremus rhinorhynchos* | 24.6 | 4.5 |
| Blenniidae | *Atrosalarias holomelas* | 25.4 | 2.0 |
| Blenniidae | *Ecsenius namiyei* | 26.3 | 2.0 |
| Blenniidae | *Ecsenius lineatus* | 24.6 | 2.0 |
| Caesionidae | *Caesio caerulaurea* | 24.7 | 3.4 |
| Caesionidae | *Pterocaesio marri* | 25.2 | 3.4 |
| Caesionidae | *Pterocaesio digramma* | 24.7 | 3.4 |
| Callionymidae | *Dactylopus dactylopus* | 24.5 | 3.4 |
| Carangidae | *Seriola dumerili* | 16.9 | 4.5 |
| Carangidae | *Selaroides leptolepis* | 25.1 | 3.8 |
| Chaetodontidae | *Chaetodon adiergastos* | 27.5 | 3.5 |
| Chaetodontidae | *Chaetodon lunula* | 23.7 | 3.7 |
| Chaetodontidae | *Chaetodon argentatus* | 24.2 | 2.0 |
| Chaetodontidae | *Chaetodon punctatofasciatus* | 26.0 | 3.4 |
| Chaetodontidae | *Coradion chrysozonus* | 25.2 | 2.8 |
| Chaetodontidae | *Heniochus monoceros* | 24.7 | 3.5 |
| Chaetodontidae | *Chaetodon bennetti* | 25.2 | 3.1 |
| Chaetodontidae | *Chaetodon ornatissimus* | 24.8 | 3.3 |
| Chaetodontidae | *Chaetodon plebeius* | 25.4 | 3.3 |
| Chaetodontidae | *Chaetodon ephippium* | 25.0 | 3.0 |
| Chaetodontidae | *Coradion altivelis* | 23.5 | 2.7 |
| Chaetodontidae | *Chaetodon lineolatus* | 23.7 | 3.4 |
| Chaetodontidae | *Heniochus acuminatus* | 23.6 | 3.5 |
| Chaetodontidae | *Chaetodon vagabundus* | 24.7 | 2.9 |
| Chaetodontidae | *Chaetodon melannotus* | 24.6 | 4.4 |
| Chaetodontidae | *Chaetodon trifascialis* | 24.9 | 3.3 |
| Chaetodontidae | *Chaetodon lunulatus* | 24.7 | 3.3 |
| Chaetodontidae | *Chaetodon kleinii* | 24.7 | 2.9 |
| Chaetodontidae | *Chaetodon auriga* | 25.5 | 3.7 |
| Chaetodontidae | *Chaetodon speculum* | 25.0 | 3.6 |
| Chaetodontidae | *Chaetodon octofasciatus* | 26.0 | 2.6 |
| Chaetodontidae | *Chaetodon auripes* | 19.4 | 3.5 |
| Cirrhitidae | *Paracirrhites forsteri* | 24.5 | 4.3 |
| Cirrhitidae | *Cirrhitichthys oxycephalus* | 23.2 | 4.0 |
| Cirrhitidae | *Paracirrhites arcatus* | 24.5 | 3.6 |
| Cirrhitidae | *Cirrhitichthys falco* | 25.2 | 4.0 |
| Dasyatidae | *Taeniura meyeni* | 23.3 | 4.2 |
| Dasyatidae | *Hemitrygon bennettii* | 24.7 | 4.5 |
| Dasyatidae | *Neotrygon kuhlii* | 23.9 | 3.3 |
| Diodontidae | *Diodon hystrix* | 23.3 | 3.7 |
| Diodontidae | *Diodon holocanthus* | 21.5 | 3.9 |
| Fistulariidae | *Fistularia petimba* | 21.3 | 4.4 |
| Gerreidae | *Gerres filamentosus* | 24.8 | 3.3 |
| Gerreidae | *Gerres japonicus* | - | 3.4 |
| Gerreidae | *Gerres oyena* | 25.2 | 2.7 |
| Gobiidae | *Bathygobius fuscus* | 24.9 | 3.4 |
| Gobiidae | *Bryaninops yongei* | 24.8 | 3.2 |
| Gobiidae | *Cryptocentrus albidorsus* | 25.2 | 3.3 |
| Gobiidae | *Gnatholepis cauerensi* | 24.7 | 2.3 |
| Gobiidae | *Valenciennea helsdingenii* | 24.4 | 3.4 |
| Gobiidae | *Valenciennea puellaris* | 25.5 | 3.2 |
| Gobiidae | *Valenciennea strigata* | 25.0 | 4.0 |
| Gobiidae | *Eviota albolineata* | 25.1 | 3.1 |
| Gobiidae | *Amblygobius phalaena* | - | 3.6 |
| Gobiidae | *Valenciennea parva* | 24.6 | 3.2 |
| Gobiidae | *Istigobius decoratus* | 24.6 | 3.4 |
| Gobiidae | *Amblyeleotris wheeleri* | 25.5 | 3.4 |
| Gobiidae | *Eviota abax* | 17.1 | 3.2 |
| Gobiidae | *Istigobius campbelli* | 24.3 | 3.3 |
| Gobiidae | *Asterropteryx semipunctata* | 24.7 | 2.4 |
| Gobiidae | *Eviota sigillata* | 24.7 | 3.1 |
| Haemulidae | *Plectorhinchus chaetodonoides* | 25.5 | 3.8 |
| Haemulidae | *Plectorhinchus lineatus* | 24.4 | 3.9 |
| Haemulidae | *Plectorhinchus picus* | 24.6 | 3.9 |
| Haemulidae | *Plectorhinchus pictus* | 21.1 | 3.9 |
| Haemulidae | *Plectorhinchus lessonii* | 24.9 | 3.7 |
| Hemiscylliidae | *Chiloscyllium plagiosum* | 25.3 | 4.0 |
| Holocentridae | *Sargocentron diadema* | 24.7 | 3.4 |
| Holocentridae | *Sargocentron rubrum* | 25.0 | 3.6 |
| Kuhliidae | *Kuhlia mugil* | 24.5 | 3.8 |
| Kyphosidae | *Kyphosus cinerascens* | 24.7 | 2.9 |
| Kyphosidae | *Microcanthus strigatus* | 17.5 | 3.0 |
| Labridae | *Coris aygula* | 24.6 | 3.7 |
| Labridae | *Halichoeres orientalis* | 21.4 | 3.3 |
| Labridae | *Halichoeres trimaculatus* | 24.7 | 3.5 |
| Labridae | *Hologymnosus annulatus* | 24.7 | 4.2 |
| Labridae | *Hologymnosus rhodonotus* | 20.6 | 3.9 |
| Labridae | *Oxycheilinus digramma* | 25.4 | 3.7 |
| Labridae | *Anampses geographicus* | 24.6 | 3.5 |
| Labridae | *Epibulus insidiator* | 24.7 | 4.0 |
| Labridae | *Labroides bicolor* | 24.7 | 4.0 |
| Labridae | *Oxycheilinus celebicus* | 25.4 | 3.8 |
| Labridae | *Paracheilinus carpenteri* | 23.9 | 3.4 |
| Labridae | *Stethojulis strigiventer* | 24.9 | 3.1 |
| Labridae | *Bodianus axillaris* | 24.4 | 3.4 |
| Labridae | *Halichoeres marginatus* | 25.0 | 3.2 |
| Labridae | *Novaculichthys taeniourus* | 24.9 | 3.3 |
| Labridae | *Pteragogus flagellifer* | 21.5 | 3.5 |
| Labridae | *Cheilio inermis* | 24.4 | 3.5 |
| Labridae | *Halichoeres argus* | 25.1 | 3.4 |
| Labridae | *Halichoeres margaritaceus* | 24.7 | 3.7 |
| Labridae | *Labrichthys unilineatus* | 26.6 | 3.3 |
| Labridae | *Bodianus diana* | 24.9 | 3.4 |
| Labridae | *Pseudocheilinus evanidus* | 24.9 | 3.5 |
| Labridae | *Anampses twistii* | 24.9 | 3.5 |
| Labridae | *Anampses meleagrides* | 24.3 | 2.5 |
| Labridae | *Hemigymnus fasciatus* | 24.9 | 3.5 |
| Labridae | *Thalassoma purpureum* | 24.6 | 3.8 |
| Labridae | *Bodianus mesothorax* | 26.5 | 3.2 |
| Labridae | *Labropsis manabei* | 22.9 | 3.3 |
| Labridae | *Thalassoma jansenii* | 23.6 | 3.1 |
| Labridae | *Anampses melanurus* | 25.3 | 3.4 |
| Labridae | *Macropharyngodon meleagris* | 24.7 | 3.1 |
| Labridae | *Pseudocheilinus hexataenia* | 24.5 | 3.2 |
| Labridae | *Choerodon azurio* | 20.4 | 3.5 |
| Labridae | *Halichoeres hortulanus* | 24.8 | 3.4 |
| Labridae | *Oxycheilinus unifasciatus* | 23.6 | 4.1 |
| Labridae | *Cheilinus chlorourus* | 25.0 | 3.5 |
| Labridae | *Hologymnosus doliatus* | 24.7 | 3.8 |
| Labridae | *Hemigymnus melapterus* | 24.6 | 3.6 |
| Labridae | *Halichoeres biocellatus* | 25.6 | 3.4 |
| Labridae | *Macropharyngodon negrosensis* | 25.2 | 3.5 |
| Labridae | *Oxycheilinus bimaculatus* | 25.2 | 3.5 |
| Labridae | *Anampses caeruleopunctatus* | 24.7 | 3.4 |
| Labridae | *Coris dorsomacula* | 22.4 | 3.5 |
| Labridae | *Cheilinus trilobatus* | 25.2 | 3.9 |
| Labridae | *Parajulis poecilepterus* | - | 3.6 |
| Labridae | *Pseudolabrus japonicus* | 17.6 | 3.1 |
| Labridae | *Coris gaimard* | 24.3 | 3.5 |
| Labridae | *Choerodon schoenleinii* | 24.5 | 3.4 |
| Labridae | *Suezichthys gracilis* | 19.5 | 3.3 |
| Labridae | *Stethojulis trilineata* | 26.7 | 3.2 |
| Labridae | *Gomphosus varius* | 24.5 | 3.7 |
| Labridae | *Thalassoma quinquevittatum* | 25.0 | 3.6 |
| Labridae | *Thalassoma lutescens* | 24.3 | 3.7 |
| Labridae | *Cirrhilabrus cyanopleura* | 26.3 | 3.4 |
| Labridae | *Thalassoma hardwicke* | 24.8 | 3.5 |
| Labridae | *Labroides dimidiatus* | 24.6 | 3.5 |
| Labridae | *Thalassoma amblycephalum* | 24.8 | 3.1 |
| Labridae | *Stethojulis bandanensis* | 24.5 | 3.2 |
| Labridae | *Stethojulis terina* | 18.4 | 3.5 |
| Labridae | *Thalassoma lunare* | 24.6 | 3.5 |
| Labridae | *Halichoeres nebulosus* | 24.7 | 3.3 |
| Labridae | *Halichoeres melanochir* | 24.7 | 3.3 |
| Lethrinidae | *Lethrinus harak* | 25.7 | 3.6 |
| Lethrinidae | *Lethrinus obsoletus* | 26.1 | 3.9 |
| Lethrinidae | *Lethrinus nebulosus* | 24.2 | 3.8 |
| Lutjanidae | *Lutjanus argentimaculatus* | 24.3 | 3.6 |
| Lutjanidae | *Lutjanus quinquelineatus* | 24.7 | 3.7 |
| Lutjanidae | *Lutjanus stellatus* | - | 4.0 |
| Lutjanidae | *Lutjanus fulvus* | 24.6 | 3.6 |
| Lutjanidae | *Lutjanus decussatus* | 27.4 | 4.0 |
| Lutjanidae | *Lutjanus gibbus* | 24.5 | 4.1 |
| Lutjanidae | *Lutjanus kasmira* | 19.1 | 3.9 |
| Lutjanidae | *Lutjanus bohar* | 24.5 | 4.3 |
| Lutjanidae | *Lutjanus russellii* | 24.4 | 4.1 |
| Lutjanidae | *Lutjanus vitta* | 25.1 | 4.0 |
| Lutjanidae | *Lutjanus monostigma* | 24.8 | 4.3 |
| Lutjanidae | *Lutjanus johnii* | 25.0 | 4.2 |
| Lutjanidae | *Lutjanus fulviflamma* | 24.7 | 3.8 |
| Microdesmidae | *Nemateleotris magnifica* | 24.7 | 3.1 |
| Microdesmidae | *Ptereleotris zebra* | 25.0 | 3.4 |
| Microdesmidae | *Ptereleotris heteroptera* | 24.5 | 3.4 |
| Microdesmidae | *Ptereleotris evides* | 25.8 | 3.4 |
| Microdesmidae | *Ptereleotris microlepis* | 25.0 | 3.4 |
| Monacanthidae | *Pervagor janthinosoma* | 23.7 | 2.9 |
| Monacanthidae | *Paraluteres prionurus* | 25.2 | 2.7 |
| Monacanthidae | *Monacanthus chinensis* | 21.2 | 2.4 |
| Mullidae | *Mulloidichthys flavolineatus* | 24.5 | 3.8 |
| Mullidae | *Mulloidichthys vanicolensis* | 24.3 | 3.6 |
| Mullidae | *Parupeneus barberinoides* | 24.7 | 3.4 |
| Mullidae | *Parupeneus barberinus* | 24.6 | 3.4 |
| Mullidae | *Parupeneus trifasciatus* | 23.6 | 3.5 |
| Mullidae | *Parupeneus indicus* | 26.2 | 3.5 |
| Mullidae | *Upeneus tragula* | 24.8 | 3.6 |
| Mullidae | *Parupeneus ciliatus* | 24.3 | 3.5 |
| Mullidae | *Parupeneus multifasciatus* | 23.8 | 3.5 |
| Muraenidae | *Echidna nebulosa* | 24.9 | 4.0 |
| Muraenidae | *Gymnothorax eurostus* | 21.5 | 4.1 |
| Muraenidae | *Gymnothorax meleagris* | 24.5 | 4.0 |
| Muraenidae | *Gymnothorax favagineus* | 24.5 | 4.2 |
| Nemipteridae | *Scolopsis monogramma* | 24.9 | 3.5 |
| Nemipteridae | *Scolopsis lineata* | 26.0 | 3.8 |
| Nemipteridae | *Scolopsis bilineata* | 25.1 | 3.6 |
| Nemipteridae | *Scolopsis vosmeri* | 25.4 | 3.5 |
| Ostraciidae | *Ostracion cubicus* | 24.5 | 3.4 |
| Pempheridae | *Pempheris oualensis* | 25.5 | 3.6 |
| Pinguipedidae | *Parapercis snyderi* | 20.5 | 3.4 |
| Pinguipedidae | *Parapercis xanthozona* | 24.7 | 3.6 |
| Pinguipedidae | *Parapercis millepunctata* | 24.8 | 3.5 |
| Pinguipedidae | *Parapercis tetracantha* | 25.5 | 3.6 |
| Pinguipedidae | *Parapercis pacifica* | 25.1 | 3.8 |
| Pinguipedidae | *Parapercis clathrata* | 26.1 | 3.6 |
| Pomacanthidae | *Apolemichthys trimaculatus* | 25.0 | 2.6 |
| Pomacanthidae | *Centropyge tibicen* | 24.5 | 2.8 |
| Pomacanthidae | *Pomacanthus imperator* | 24.6 | 2.7 |
| Pomacanthidae | *Centropyge vrolikii* | 25.6 | 2.8 |
| Pomacanthidae | *Pomacanthus semicirculatus* | 24.7 | 2.7 |
| Pomacanthidae | *Chaetodontoplus septentrionalis* | 21.2 | 2.7 |
| Pomacentridae | *Abudefduf septemfasciatus* | 25.6 | 3.0 |
| Pomacentridae | *Neoglyphidodon melas* | 25.1 | 3.4 |
| Pomacentridae | *Pomacentrus moluccensis* | 25.1 | 2.4 |
| Pomacentridae | *Pomacentrus philippinus* | 25.3 | 2.7 |
| Pomacentridae | *Amblyglyphidodon curacao* | 24.7 | 2.6 |
| Pomacentridae | *Neoglyphidodon nigroris* | 25.2 | 3.0 |
| Pomacentridae | *Plectroglyphidodon lacrymatus* | 24.7 | 2.2 |
| Pomacentridae | *Pomacentrus vaiuli* | 25.4 | 3.1 |
| Pomacentridae | *Chromis atripectoralis* | 25.0 | 3.1 |
| Pomacentridae | *Chromis cinerascens* | 26.8 | 2.7 |
| Pomacentridae | *Plectroglyphidodon dickii* | 25.0 | 3.7 |
| Pomacentridae | *Abudefduf sordidus* | 24.7 | 2.9 |
| Pomacentridae | *Chrysiptera cyanea* | 25.9 | 2.5 |
| Pomacentridae | *Neopomacentrus taeniurus* | 26.8 | 3.4 |
| Pomacentridae | *Stegastes apicalis* | 24.4 | - |
| Pomacentridae | *Chrysiptera rex* | 25.4 | 2.5 |
| Pomacentridae | *Chromis notata* | 18.9 | 3.4 |
| Pomacentridae | *Pomacentrus bankanensis* | 25.5 | 2.7 |
| Pomacentridae | *Chromis lepidolepis* | 24.8 | 3.4 |
| Pomacentridae | *Dascyllus reticulatus* | 24.6 | 3.1 |
| Pomacentridae | *Chromis margaritifer* | 25.0 | 3.0 |
| Pomacentridae | *Amphiprion clarkii* | 25.3 | 2.9 |
| Pomacentridae | *Dascyllus trimaculatus* | 24.6 | 2.8 |
| Pomacentridae | *Abudefduf vaigiensis* | 21.9 | 2.6 |
| Pomacentridae | *Abudefduf sexfasciatus* | 24.7 | 2.7 |
| Pomacentridae | *Abudefduf bengalensis* | 25.0 | 3.1 |
| Pomacentridae | *Neopomacentrus cyanomos* | 25.2 | 3.4 |
| Pomacentridae | *Chromis fumea* | 18.8 | 3.4 |
| Pomacentridae | *Stegastes fasciolatus* | 18.5 | 2.2 |
| Pomacentridae | *Pomacentrus coelestis* | 24.9 | 3.2 |
| Priacanthidae | *Priacanthus hamrur* | 19.1 | 3.6 |
| Pseudochromidae | *Labracinus cyclophthalmus* | 26.0 | 3.9 |
| Rachycentridae | *Rachycentron canadum* | 8.1 | 4.0 |
| Scaridae | *Leptoscarus vaigiensis* | 24.3 | 2.0 |
| Scaridae | *Scarus ovifrons* | 18.8 | - |
| Scaridae | *Cetoscarus bicolor* | 24.9 | 2.0 |
| Scaridae | *Scarus oviceps* | 25.0 | 2.0 |
| Scaridae | *Scarus prasiognathos* | 26.1 | 2.0 |
| Scaridae | *Scarus psittacus* | 24.7 | 2.0 |
| Scaridae | *Scarus festivus* | 25.1 | 2.0 |
| Scaridae | *Scarus hypselopterus* | 25.4 | - |
| Scaridae | *Scarus schlegeli* | 24.5 | 2.0 |
| Scaridae | *Scarus forsteni* | 25.0 | 2.0 |
| Scaridae | *Scarus dimidiatus* | 25.2 | 2.0 |
| Scaridae | *Scarus frenatus* | 24.7 | 2.0 |
| Scaridae | *Scarus niger* | 24.9 | 2.0 |
| Scaridae | *Chlorurus sordidus* | 24.6 | 2.6 |
| Scaridae | *Scarus ghobban* | 24.5 | 2.0 |
| Scaridae | *Chlorurus gibbus* | 24.6 | 2.0 |
| Scaridae | *Scarus rubroviolaceus* | 24.9 | 2.0 |
| Scaridae | *Scarus rivulatus* | 24.8 | 2.0 |
| Scorpaenidae | *Dendrochirus bellus* | 15.3 | 3.8 |
| Scorpaenidae | *Pterois antennata* | 24.6 | 3.6 |
| Scorpaenidae | *Pterois lunulata* | 5.7 | 3.7 |
| Scorpaenidae | *Scorpaenopsis diabolus* | 24.6 | 4.2 |
| Scorpaenidae | *Sebastapistes strongia* | 24.4 | 3.8 |
| Scorpaenidae | *Scorpaenopsis cirrosa* | 18.2 | 4.2 |
| Scorpaenidae | *Dendrochirus zebra* | 24.3 | 4.0 |
| Scorpaenidae | *Pterois volitans* | 22.4 | 4.4 |
| Sebastidae | *Sebastiscus marmoratus* | 18.2 | 3.7 |
| Serranidae | *Epinephelus coeruleopunctatus* | 24.6 | 3.7 |
| Serranidae | *Cephalopholis miniata* | 23.7 | 4.3 |
| Serranidae | *Cephalopholis urodeta* | 25.2 | 4.0 |
| Serranidae | *Epinephelus areolatus* | 22.4 | 3.7 |
| Serranidae | *Epinephelus hexagonatus* | 25.1 | 4.1 |
| Serranidae | *Variola louti* | 20.1 | 4.3 |
| Serranidae | *Epinephelus malabaricus* | 24.3 | 4.2 |
| Serranidae | *Epinephelus trimaculatus* | 18.7 | 4.0 |
| Serranidae | *Grammistes sexlineatus* | 25.2 | 4.0 |
| Serranidae | *Cromileptes altivelis* | 25.0 | 4.5 |
| Serranidae | *Epinephelus merra* | 24.6 | 3.8 |
| Serranidae | *Pseudanthias squamipinnis* | 24.3 | 3.4 |
| Serranidae | *Diploprion bifasciatum* | 24.6 | 4.0 |
| Serranidae | *Epinephelus awoara* | 16.2 | 3.6 |
| Serranidae | *Cephalopholis boenak* | 24.7 | 4.1 |
| Serranidae | *Plectropomus leopardus* | 24.7 | 4.4 |
| Serranidae | *Epinephelus quoyanus* | 24.5 | 4.0 |
| Siganidae | *Siganus spinus* | 24.6 | 2.0 |
| Siganidae | *Siganus fuscescens* | 22.6 | 2.0 |
| Sphyraenidae | *Sphyraena flavicauda* | 24.3 | 3.8 |
| Synanceiidae | *Synanceia verrucosa* | 24.9 | 4.2 |
| Synodontidae | *Trachinocephalus myops* | 18.9 | 4.4 |
| Synodontidae | *Saurida gracilis* | 24.0 | 4.2 |
| Synodontidae | *Synodus variegatus* | 24.2 | 4.2 |
| Tetraodontidae | *Arothron nigropunctatus* | 24.7 | 3.4 |
| Tetraodontidae | *Canthigaster solandri* | 26.1 | 3.0 |
| Tetraodontidae | *Arothron hispidus* | 23.7 | 3.2 |
| Tetraodontidae | *Canthigaster valentini* | 24.6 | 2.8 |
| Tripterygiidae | *Enneapterygius hsiojenae* | - | 3.1 |
| Tripterygiidae | *Helcogramma striata* | 25.1 | 3.4 |
| Zanclidae | *Zanclus cornutus* | 20.0 | 2.5 |
